# Sub 3-Hour Marathon Runners for Five Consecutive Decades Demonstrate a Reduced Age-Related Decline in Performance

**DOI:** 10.3389/fphys.2021.649282

**Published:** 2021-02-23

**Authors:** Romuald Lepers, Amby Burfoot, Paul J. Stapley

**Affiliations:** ^1^CAPS UMR1093, Institut National de la Santé et de la Recherche Médicale (INSERM), Faculté des Sciences du Sport, UFR STAPS, Université de Bourgogne-Franche Comté, Dijon, France; ^2^PodiumRunner, Boulder, CO, United States; ^3^Neural Control of Movement Laboratory, School of Medicine, Faculty of Science, Medicine and Health, University of Wollongong, Wollongong, NSW, Australia

**Keywords:** marathon athletes, masters athletes, master athlete, aging athletes, elderly athletes

## Abstract

Estimation of the age-related decline in athletic performance by analyzing age-group world record performances presents an inherent limitation because the records generally belong to different individuals. Longitudinal studies describing the changes in performance with advancing age for the same individuals with a consistent training regimen are more appropriate to determine age-related changes in performance. The aim of this longitudinal study was to examine the age-related decline in running performance of sub 3-h marathoners for five consecutive calendar decades. The best marathon performances for each decade from the 1970s to the 2010s were analyzed for 40 sub 3-h runners (39 males and 1 female). The cohort mean personal best performance was 2 h 23 min ± 9 min at an age of 28.6 ± 4.7 years. The mean difference in age between the first and the last sub 3-h marathon races was 32.9 ± 1.6 years. The time difference in marathon performance between the personal best and the worst performance during the 5th decade was 26 ± 9 min, corresponding to a mean increase of 1 min 4 s per year, i.e., a decrease in running speed of 0.67 ± 0.29% per year. These results suggest that with consistent training and racing regimens, it is possible to limit the age-related decline in marathon performance to less than 7% per decade at least until 60 years of age. Further studies are required to verify if such a low rate of age-related decline in endurance performance could be maintained after 60 years of age.

## Introduction

The age-related decline in endurance running performance has been extensively studied, especially for the marathon (Stones and Kozma, 1980; Jokl et al., 2004; Leyk et al., 2007; Trappe, 2007; Lepers and Cattagni, 2012; Knechtle et al., 2014, 2018; Lepers and Stapley, 2016). Fastest running marathon performances are generally observed at 25–35 years of age for both elite and non-elite runners (Hunter et al., 2011; Nikolaidis et al., 2019). With advancing age, marathon running performance decreases linearly until 75–80 years old and exponentially thereafter (Rittweger et al., 2009; Lepers and Cattagni, 2018). The reasons for this accelerated decline or “breakpoint” may be explained by different factors such as a reduction of the pool of older competitors, a decrease in the amount and intensity of training, or a reduction of integrative physiological capacity despite the maintenance of training level (Lazarus and Harridge, 2017).

Estimation of the age-related decline in athletic performance is generally assessed by analyzing world record performances for each age-group category. For the marathon, the under 40 years old world record time is currently 2:01:39, i.e., 5.78 m.s^−1^ (set by the Kenyan Eliud Kipchoge at 34 years old), while that of the 60–64 year category is 2:36:30, i.e., 4.49 m.s^−1^ (set by the Japanese athlete, Yoshihisa Hosaka at 60 years old).^1^ A comparison of these two athletes’ performances reveals a reduction in running speed of 8.5% per decade. However, estimating age-related decline in running performance using methodologies of direct comparison of performance across age groups may be problematic or limited in its ability to draw conclusions. Indeed, world record holders are often different in their athletic background, demographic (nationality), and even somatotype. To overcome these limitations, longitudinal studies describing the changes in performance with advancing age for the same individuals, who were consistent in their training habits, may be more appropriate to determine the age-related change in performance.

Unfortunately, reports of longitudinal studies of individual athletes over a long time period are scarce. Some case studies have described the age-related decline in running performance for runners older than 60 years, confirming a larger decline after 80 years, but their training regime at younger ages were not consistent enough and thus the information regarding their age-related decline in performance is limited (Knechtle et al., 2010; Lepers and Cattagni, 2018). Interestingly, it has recently been shown that a 60-year old former Olympic marathoner had managed to limit his age-related decline in performance to less than 5% per decade (Lepers et al., 2020; Louis et al., 2020). This example clearly shows that age-related decline in performance for an individual can be different, in this case lower, than a prediction based on world records at different ages.

Outdoor non-stadia running races such as marathons have grown worldwide since the late 1970’s. There are some unique individuals who ran their first marathon in late 1970’s and have kept running marathons until 2010’s, therefore, over five consecutive calendar decades (5D). Moreover, and somewhat impressively, some of them have been able to run a sub 3-h marathon (S3) all during this period. They have, therefore, been named the “5DS3” runners, i.e., sub 3-h marathoners for the five consecutive calendar decades (between the 1970s and the end of the 2010s; Burfoot, 2020a). These athletes with their level of performance and their regularity over the years despite their advancing age, represent an interesting experimental model to analyze the age-related decline in endurance running performance. In the present study, based on a longitudinal analysis, we examined the age-related decline in marathon performance of these sub 3-h runners over the five consecutive calendar decades.

## Materials And Methods

Data were collected from the website of the Association of Road Race Statisticians^2^ that established a list of 41 runners (40 males and 1 female) who ran a sub-3 h marathon of the five consecutive calendar decades (1970s, 1980s, 1990s, 2000s, and 2010s). For each runner, the best performances for each calendar decade were considered. The races with no gun time available, excessive continuous descents in altitude during races, or courses that were shorter than the regular distance were not considered in our analysis. One runner was excluded because four of his five performances were ineligible. Mean marathon running speed was calculated. Ages of personal best and worst performances were identified for each runner based on available data. Percent change was calculated to quantify the relative change in marathon performance between the personal best and the worst performance of each runner.

## Results

**Figure 1:**
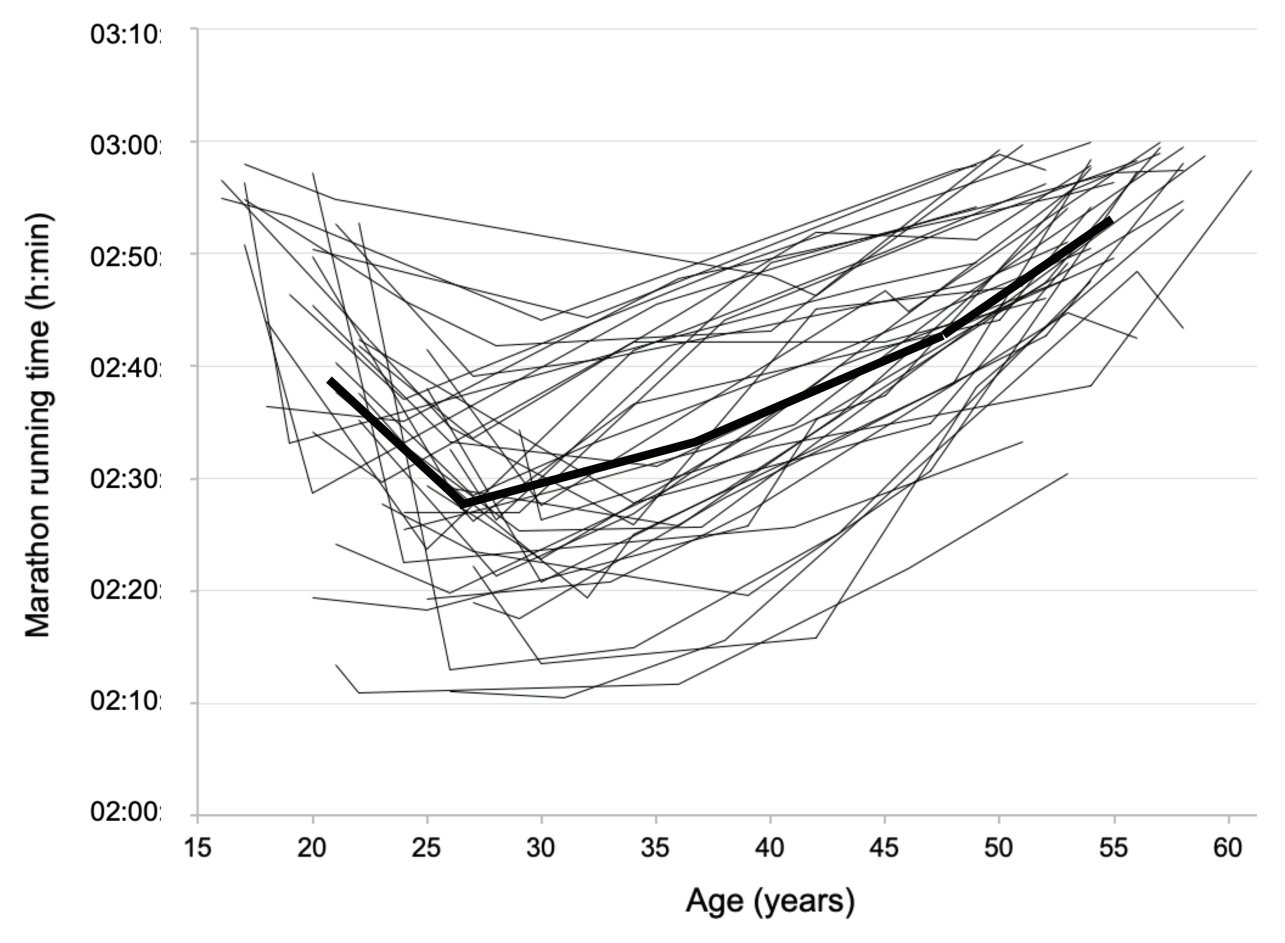
Marathon time performances of the 5DS3 runners (*n* = 40) plotted against increasing age during the five consecutive calendar decades (from 1970s to 2010s). The bold line represents the mean trace of all athletes.

**Figure 2:**
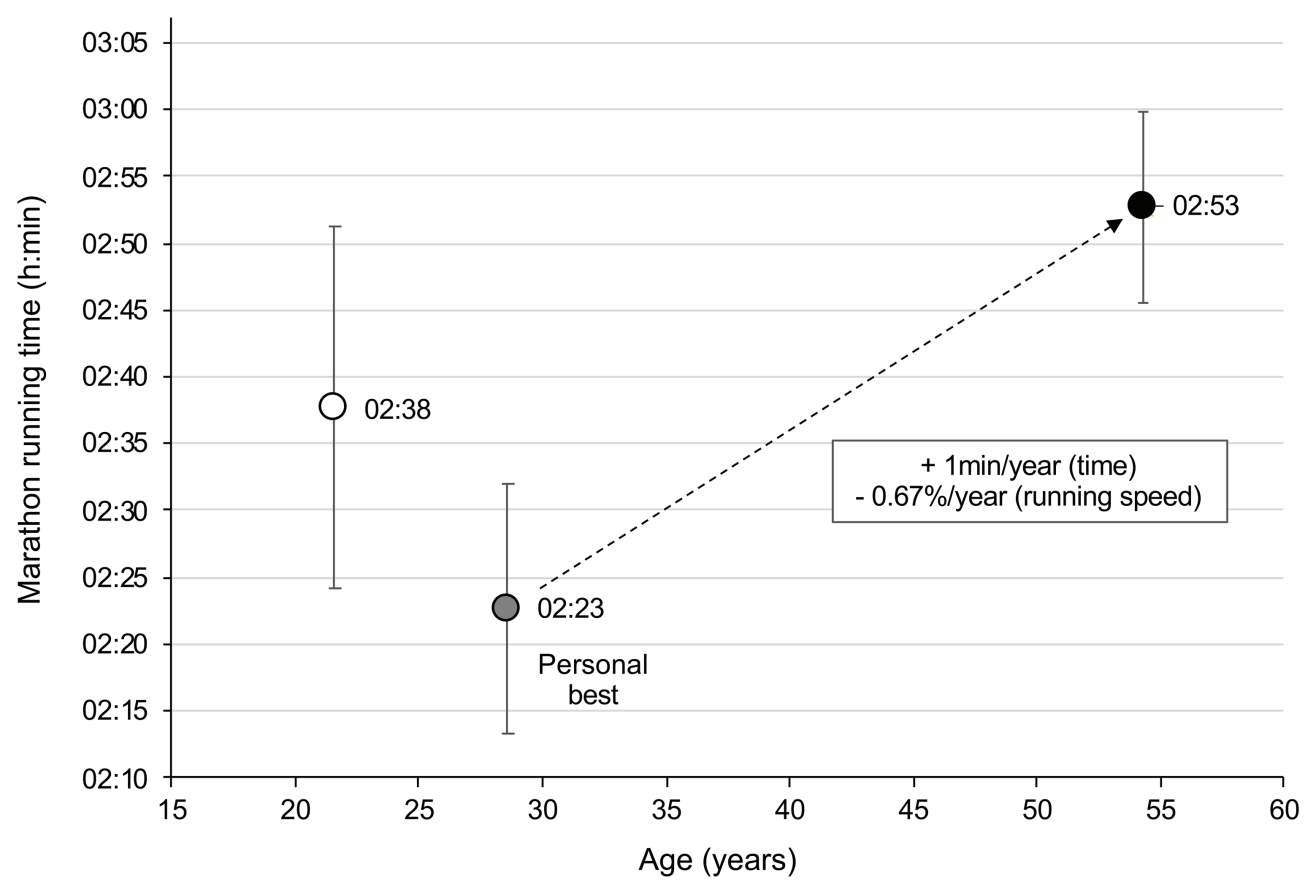
Mean (+/−1 SD) changes in marathon running performance of the 5DS3 runners (*n* = 40) plotted against increasing age between their first race and their last race.

The best marathon performances of the 5DS3 runners for each five consecutive decades are presented in Table 1. The mean difference in age between the first and the last marathon races was 32.9 ± 1.6 years. Table 2 shows that 73% of 5DS3 runners achieved their personal best performance between 25 and 34 years old. Marathon time performances of the 5DS3 runners during the five consecutive calendar decades are shown in Figure 1. Eleven athletes ran at least one marathon in under 2 h 20 min and three athletes ran at least one marathon in under 2 h 15 min. The mean athletes’ personal best performance was 2 h 23 min ± 9 min at a mean age of 28.6 ± 4.7 years. The mean time difference in marathon performance between the personal best and the worst performance during the 5th decade was 26 min ± 9 min, corresponding to a mean increase of 1 min 4 s per year, i.e., a decrease in running speed of 0.67 ± 0.29% per year (see Figure 2).

**Table 1:** Mean age and marathon time performance of the 5DS3 runners (*n* = 40) during the five consecutive calendar decades.

Decades	Age (years)	Marathon time (h:min)
1970–1979	21.6 ± 3.4	2:38 ± 13
1980–1989	26.9 ± 3.7	2:27 ± 10
1990–1999	36.9 ± 3.7	2:33 ± 11
2000–2009	47.2 ± 4.2	2:43 ± 9
2010–2019	54.3 ± 3.1	2:53 ± 7

Values are mean ± SD.

**Table 2:** Number (and percentage of the cohort) of runners who ran their personal best times in each age-group category.

Age group	Number	%
<20 years	1	3
20–24 years	6	15
25–29 years	17	43
30–34 years	12	30
35–39 years	3	8
40–44 years	1	3

## Discussion

The aim of this longitudinal study was to analyze the age-related decline in running performance of sub 3-h marathoners for the five consecutive calendar decades. Our results have shown that these well-trained athletes were able to limit their decrease in marathon running speed to less than 0.7% per year for about 30 years following their personal best records. The longitudinal data presented in this study are similar to previous cross-sectional data suggesting that the age-related decline in endurance running performance appears to be 6–7% per decade for males up to their late 1950s (Joyner, 1993).

This study is unique as it used data from an elite set of marathoners. According to the website marastats.com that collected and analyzed finish times for 3 million marathon runners around the world over the past two decades, only 4% of male runners and 1% of female runners finish a marathon in under 3 h (Leyk et al., 2007). Being able to run a sub 3-h marathon for the five consecutive calendar decades is, therefore, exceptional and, therefore, explains the relatively low “*n*” (40) used in this study.

The 5DS3 athletes analyzed in this study reached their personal best marathon performance at a mean age of ~29 years (73% of them reached it between 25 and 34 years) which is accordance with previous findings of elite marathoners (Hunter et al., 2011). The age of peak marathon performance has previously been studied using different sampling approaches (e.g., elite athletes, top age-groupers, all finishers, etc.) and has been estimated to occur between 25 and 35 years. It seems, however, that age of peak marathon performance is lower for male elite athletes (~29 years) than for male amateur athletes (~34 years; Hunter et al., 2011; Nikolaidis et al., 2019). It has also been shown that the age of peak endurance performance for elite athletes has increased over the past four or five decades for ultra-running and Ironman triathlon for both males and females (Hoffman and Wegelin, 2009; Gallmaan et al., 2014) but it remains to be verified for marathon running.

Estimation of the age-related decline in marathon performance is generally assessed by analyzing world record performances for each age-group category (Leyk et al., 2007; Santos-Lozano et al., 2015). For example, Santos-Lozano et al. (2015) showed that the effect of aging differed between the fastest and slowest marathoners. Until 60 years old, the age-related decline in the fastest runners, ranked in the highest (90th) percentiles of performance relative to their age category, was less pronounced than those of lowest runners (~8 vs. ~10% per decade). A study of Stones (2019) also confirmed that marathon performance times show less marked age declines among faster than slower runners. A greater retention of faster racing times in the best masters runners could be a consequence of a prolonged continuation of their effective training practices.

Consistency in training with advancing age seems not always to be required to limit age-related declines in performance. Indeed, a recent case report showed that despite a 16-year break in training (from 32 to 48 years), a 59-year old former Olympian marathoner – who established a new single age marathon World record – managed to limit his age-related decline in performance to ~5% per decade (Lepers et al., 2020). However, it should be noted that since re-starting training after this long break, this elite master athlete maintained a high training volume, running as much as 160 km per week during specific training periods for a marathon race.

Age-related declines in endurance performance are dependent on the mode of locomotion. Several studies have shown a smaller age-related decline in cycling performance than in running and swimming performances (Bernard et al., 2010; Lepers et al., 2010, 2013, 2018; Lepers and Stapley, 2016a). Several hypotheses based on debilitating injuries, biomechanical, physiological, and training considerations have been proposed to explain the smaller decline in cycling performance with advanced age but the reasons for such discipline-specificity remains unclear. Masters runners are more likely to experience multiple injuries and with different profiles compared to younger runners (McKean et al., 2006; Willy and Paquette 2019). Based on these observations, we could expect that well-trained masters cyclists could reduce even more the age-related decline in performance compared to well-trained masters runners. Anecdotally, the performance of the centenarian cyclist Robert Marchand during the hour record in track cycling, when it was compared with the hour world record, corresponded to an age-related decline in performance of less than 8% per decade for more than six decades (Lepers et al., 2016b). Longitudinal studies describing the changes in cycling and swimming performance with advancing age for the same individuals are required in the future.

To date, among the 40 athletes included in this study, only four athletes have run a sub 3-h marathon in their sixth consecutive calendar decade (i.e., 2020s) and become 6DS3 runners (Burfoot, 2020b). The one athlete who has the longest span between sub 3-h marathons (43 years and 77 days) achieved his personal best at 25 years old (2 h 18 min) and ran 2 h 54 min at 62 years. His average decrease in performance was 0.69% per year over the 37 years period that corresponds to the average decline in performance of the group of the “5DS3” runners.

Although aging is associated with a progressive decline in aerobic capacity such as maximal oxygen uptake (VO_2max_), maintaining high levels of physical exercise along the lifespan attenuates a decline in age-related aerobic capacity (Valenzuela et al., 2020). A reduced age-related decline in marathon performance is associated with a conservation of a high cardiorespiratory capacity (i.e., VO_2max_) associated with a very good specific endurance capacity at marathon pace with advancing age. The ability to extract O_2_ (i.e., diffusive O_2_ transport from blood to mitochondria) to be utilized by mitochondria is an essential step for an optimal cardiorespiratory capacity. Distefano et al. (2017) showed that chronological aging in recreationally active individuals did not affect mitochondrial related gene and protein expression. However, high levels of physical activity, even in advancing age, result in greater mitochondrial-related gene expression and protein content compared to young and age matched untrained individuals (Broskey et al., 2015; Joanisse et al., 2020), supporting the notion that skeletal muscle retains the ability to positively respond to stimuli even in advancing age.

It has been recently been shown through two studies that high-level masters runners were able to sustain a running velocity eliciting more than 90% of VO_2max_ during the marathon (Robinson et al., 2019; Louis et al., 2020). These observations suggest that compared to young runners, masters runners might be able to run closer to their VO_2max_ for the duration of the marathon (Allen et al., 1985). Running economy is also important to running performance, but the effect of age on running economy on the trained runner is unclear. Running economy has been found to decrease with aging in Olympic-caliber running athletes when they stop competitions (Everman et al., 2018); however, it seems that high level master athletes are able to maintain a high running economy (i.e., close to 210 ml.kg^−1^.km^−1^) at the marathon pace (Lepers et al., 2020; Louis et al., 2020). Beck et al. (2016) showed that non-elite runners older than 65 years ran at a given speed with similar oxygen consumption as young runners, but because their VO_2max_ was lower, it corresponded to a higher percentage of the VO_2max_. Interestingly, these older runners maintained a good running economy despite biomechanical differences. Indeed, their elicited ground reaction forces and stride kinematics differed from those of young runners and their leg stiffness was 10–20% lower than that of young runners. The mechanisms responsible for a possible alteration of running economy with aging are not yet clearly identified. It could be related to a decrease in efficiency of locomotor muscles but also of the cardiovascular/pulmonary function. For example, an increase in oxygen cost of breathing during exercise might increase whole body oxygen consumption at a given speed even if muscle oxygen consumption does not change (Joyner, 1993).

In conclusion, these results suggest that with consistent training and racing regimens, it is possible to limit the age-related decline in marathon performance to less than 7% per decade at least until 60-years-old. 5DS3 runners represent an interesting model of master athletes that retain high levels of performance over time; however, greater investigations are required to fully understand why and how some master athletes retain “elite status” over prolonged historical periods. Additional studies should also verify if such a low rate of age-related decline in endurance performance could be maintained after 60 years of age.

## Data Availability Statement

The raw data supporting the conclusions of this article will be made available by the authors, without undue reservation.

## Author Contributions

All of the listed authors (RL, AB, and PS) contributed to writing the manuscript, suggesting improvements to the manuscript, and reviewing the manuscript. All the listed authors (RL, AB, and PS) approved the final version of the manuscript.

### Conflict of Interest

The authors declare that the research was conducted in the absence of any commercial or financial relationships that could be construed as a potential conflict of interest.

## Footnotes

^1^https://world-masters-athletics.com

^2^https://www.arrs.run/TR_5Decades.htm
